# The Key Role of the WNT/β-Catenin Pathway in Metabolic Reprogramming in Cancers under Normoxic Conditions

**DOI:** 10.3390/cancers13215557

**Published:** 2021-11-05

**Authors:** Alexandre Vallée, Yves Lecarpentier, Jean-Noël Vallée

**Affiliations:** 1 Department of Clinical Research and Innovation (DRCI), Foch Hospital, 92150 Suresnes, France; 2 Centre de Recherche Clinique, Grand Hôpital de l’Est Francilien (GHEF), 6-8 Rue Saint-Fiacre, 77100 Meaux, France; yves.c.lecarpentier@gmail.com; 3 Centre Hospitalier Universitaire (CHU) Amiens Picardie, Université Picardie Jules Verne (UPJV), 80054 Amiens, France; valleejn@gmail.com; 4 Laboratoire de Mathématiques et Applications (LMA), UMR, CNRS 7348, Université de Poitiers, 86000 Poitiers, France

**Keywords:** cancer, WNT/β-catenin pathway, normoxia, Warburg effect, autophagy, lactate, aerobic glycolysis, HIF-1α, metabolic reprogramming, angiogenesis, VEGF, glutaminolysis

## Abstract

**Simple Summary:**

The canonical WNT/β-catenin pathway is upregulated in cancers and plays a major role in proliferation, invasion, apoptosis and angiogenesis. Recent studies have shown that cancer processes are involved under normoxic conditions. These findings completely change the way of approaching the study of the cancer process. In this review, we focus on the fact that, under normoxic conditions, the overstimulation of the WNT/β-catenin pathway leads to modifications in the tumor micro-environment and the activation of the Warburg effect, i.e., aerobic glycolysis, autophagy and glutaminolysis, which in turn participate in tumor growth.

**Abstract:**

The canonical WNT/β-catenin pathway is upregulated in cancers and plays a major role in proliferation, invasion, apoptosis and angiogenesis. Nuclear β-catenin accumulation is associated with cancer. Hypoxic mechanisms lead to the activation of the hypoxia-inducible factor (HIF)-1α, promoting glycolytic and energetic metabolism and angiogenesis. However, HIF-1α is degraded by the HIF prolyl hydroxylase under normoxia, conditions under which the WNT/β-catenin pathway can activate HIF-1α. This review is therefore focused on the interaction between the upregulated WNT/β-catenin pathway and the metabolic processes underlying cancer mechanisms under normoxic conditions. The WNT pathway stimulates the PI3K/Akt pathway, the STAT3 pathway and the transduction of WNT/β-catenin target genes (such as c-Myc) to activate HIF-1α activity in a hypoxia-independent manner. In cancers, stimulation of the WNT/β-catenin pathway induces many glycolytic enzymes, which in turn induce metabolic reprogramming, known as the Warburg effect or aerobic glycolysis, leading to lactate overproduction. The activation of the Wnt/β-catenin pathway induces gene transactivation via WNT target genes, c-Myc and cyclin D1, or via HIF-1α. This in turn encodes aerobic glycolysis enzymes, including glucose transporter, hexokinase 2, pyruvate kinase M2, pyruvate dehydrogenase kinase 1 and lactate dehydrogenase-A, leading to lactate production. The increase in lactate production is associated with modifications to the tumor microenvironment and tumor growth under normoxic conditions. Moreover, increased lactate production is associated with overexpression of VEGF, a key inducer of angiogenesis. Thus, under normoxic conditions, overstimulation of the WNT/β-catenin pathway leads to modifications of the tumor microenvironment and activation of the Warburg effect, autophagy and glutaminolysis, which in turn participate in tumor growth.

## 1. Introduction

Metabolic reprogramming is one of the main characteristics of cancer cell activity. Cancer metabolism reprogramming not only provides ATP (adenosine triphosphate) for cancer cells, but also essential macromolecules for its protein and nucleotide biosynthesis. Several studies have shown that cancer cell metabolic reprogramming is controlled by a number of different factors [1,2], among which are the tumor microenvironment and hypoxia. Either an inflamed microenvironment, hypoxia or both, are in part responsible for the increase in glucose uptake and lactate production levels. In parallel, expressions of several key enzymes involved in the metabolic process are activated and lead to the same results. 

Metabolic reprogramming in cancers is characterized by two processes involving autophagy and the Warburg glycolysis. Autophagy in cancers impairs mitochondrial energy homeostasis and favors inflammation and oxidative stress [3]. During tumor progression, the metabolic state of cells changes, involving both the glucose and lipid metabolism, biological oxidation and iron metabolism [4]. Metabolic reprogramming leads to several abnormalities in biochemical and biophysical functions. Cancer cells adopt a glycolysis modulation in the presence of sufficient oxygen, known as the Warburg effect or aerobic glycolysis [5]. The Warburg effect involves cell proliferation [6] and cell migration [7] but also drug resistance [8] and radioresistance [9]. The autophagy modulation and conversion of metabolism to aerobic glycolysis is a key feature of the cancer process and is associated with an aggressive clinical behavior [10]. 

The canonical WNT/β-catenin pathway modulates several signaling processes involved in tissue homeostasis. It is now well established that activation of the canonical WNT/β-catenin pathway is necessary for cancer cell survival and maintenance, making it a promising target for an anticancer therapy regimen [11]. An aberrant WNT/β-catenin pathway has been observed in numerous cancers [12,13,14,15,16] resulting in the stimulation of numerous WNT target genes involved in tumor initiation, progression and aggressiveness. These genes include c-Myc, cyclin D1, the hypoxia inducible factor-1α (HIF-1α) [17], the production of reactive oxygen species (ROSs) [18] and the activation of chronic inflammation [19]. 

In recent years, many studies have observed the increase in WNT/β-catenin signaling under normoxic conditions, leading to the enhancement of different pathways involved in the processes of cancer progression [2,20]. Thus, studies have shown the significant role of this pathway in the initiation of angiogenesis under normoxic conditions, its implication in autophagy modulation and the enhancement of the Warburg effect [17]. Nevertheless, the details of the regulatory process of metabolic reprogramming under normoxic conditions in cancer cells remain somewhat unclear. To address this point, the present review focuses on the interactions between the upregulated WNT/β-catenin pathway and the metabolic mechanisms underlying the cancer process under normoxic conditions.

## 2. Metabolic Reprogramming in Cancers: The Warburg Effect

For decades, the Warburg effect (also known as aerobic glycolysis) has been described as the sequelae of damaged mitochondria [21] and as a metabolic signature of cancer cells. At the same time, oxygen absorption rates for most cancers are determined by the availability of O_2_, which is the blood concentration of O_2_ multiplied by blood flow [22,23]. O_2_ absorption rates depend on the availability of O2 and therefore on the effectiveness of blood flow [24]. An increase in blood flow in individual tumors during hyperthermia is followed by an increase in O_2_ absorption levels [25]. Isolated tumor cells exponentially stimulate their O_2_ consumption levels as the temperature rises to a maximum of around 42 °C. The temperature coefficient Q10 obtained (also called the van ’t Hoff coefficient) is 2.3, thus clearly showing fully functional mitochondrion [25]. The concentration of O_2_ in the arterial blood feeding a tumor is a function of either the partial O_2_ arterial pressure (pO_2_), the hemoglobin content (cHb) or in combination. Modifications to each of these parameters influence the availability of oxygen as well as the rate of O2 absorption by tumors [25].

The Warburg effect still attracts considerable interest in research based on mutations in TCA (tricarboxylic acid) cycle enzymes [26] and the increase in ROSs in tumor cells due to a decline in the ROS-scavenging enzyme, superoxide dismutase-II (SOD-II), in mitochondria [27].

The aerobic glycolysis may not simply be due to an increase in the glycolytic mechanism, but also to a decrease in the activity of mitochondria. This phenomenon is stimulated by the transcription factor HIF-1α, which can be increased under both hypoxic and normoxic conditions. HIF is considered to be a major modulator of the Warburg effect. HIF-1α can decrease the mitochondrial mechanism by direct stimulation of pyruvate dehydrogenase kinase 1 (PDK-1), which in turn inhibits the PDH (pyruvate dehydrogenase). Pyruvate (the final product of glycolysis) cannot enter the TCA cycle, which results in a reduction in OXPHOS (oxidative phosphorylation), cellular oxygen consumption and ROS generation [17,28]. A second mechanism induced by HIF-1 is the decrease in the proportion of mitochondria per cell, due to the involvement of MXI1, a c-Myc antagonist, which controls the biogenesis of mitochondria. A third process, by which HIF-1 modulates the mitochondrial role, is the modification of the activity of cytochrome oxidase [28].

### 2.1. Warburg Effect, a Major Feature of Metabolic Reprogramming 

Metabolic reprogramming, a consequence of metabolism plasticity, has been observed as one of the major mechanisms in cancer processes [29]. This process is characterized by the dysregulation of the glycolytic and glutaminolytic pathways. The increase of glycolytic flow is achieved by the stimulation of the phosphatidylinositol-3-kinase/protein kinase B (PI3K/Akt) pathway and HIF-1α [20] or by other processes, such as the production of reactive oxygen species (ROSs), mutations of tumor suppressors (PTEN, p53, VHL) or oncogene activation (c-Myc, Ras, Raf). The increase of glycolytic flow can also be achieved through decreased AMPK signaling (AMP-activated protein kinase), as well as by the activation of c-Myc which stimulates glucose transporters GLUT-1 and GLUT-3 and phosphofructokinase (PFK) [30,31]. All of these alterations are implemented in the hostile tumor microenvironment (TME) under normoxic conditions, contributing to the development of the aerobic glycolysis phenotype.

In parallel with the limited supply of ATP, the Warburg effect produces antioxidant fragments (2 NADH per glucose mole) and enables the diversion of glycolytic intermediates to numerous biosynthesis pathways (NADPH, lipids and non-essential amino acids), thus supporting biosynthesis pathways as long as adequate glucose intake is controlled. These biosynthesis pathways are caused by a decrease in the role of the pyruvate kinase enzyme, which catalyzes the final stage of the Warburg effect, converting phosphoenolpyruvate into pyruvate. Aerobic glycolysis can decrease the production of ROSs [32] which, at low concentrations, can improve cancer cell survival rates [33] as long as glucose intake is maintained at the required level [34]. High glycolytic levels may involve a depletion of glucose in the TME to below physiological rates [34].

### 2.2. Warburg Effect: Release of Lactate, a Fuel for Normoxic Cancer Cells 

High rates of lactate release in TME result from increased regulation of aerobic glycolysis, and c-Myc-induced glutaminolysis, another major feature of cancer metabolic reprogramming. For increased lactate production in the Warburg effect, activation of both HIF-1α- and c-Myc increases the expression of PDK-1 and lactate dehydrogenase A (LDH-A), which converts pyruvate into lactate, contributing to the Warburg effect phenotype [35]. The increase in HIF-directed expression of PDK-1 inhibits PDH and therefore decreases the entry of pyruvate into the mitochondrial matrix and the Krebs cycle.

### 2.3. Lactates: A Fuel for Cancer Cells to Initiate Angiogenesis

The transformation of the TME occurs in order to initiate angiogenesis. To increase molecular mechanisms and cellular movements, the cellular environment is modified. High lactate release rates lead to numerous cancerous mechanisms, including microenvironment transformation [36], cellular motility [37,38], impaired immune system [39,40] and anti-apoptotic factors [41]. Lactate stimulation causes an increase in the nuclear factor-kappa B (NF-kB) pathway, which stops cell death by anti-apoptotic molecules, and initiates better cell survival in a hostile environment [42]. At the same time, lactate causes the transformation of the microenvironment, which directly activates angiogenesis [36,37,42,43,44,45,46,47,48,49].

The activation of glycolysis alters cellular pH and osmolarity by stimulating lactate production [50,51]. The TME has an acid pH [52]. In tumors, the vascular endothelial growth factor (VEGF) expression is stimulated by an acid microenvironment [53]. In cancer xenografts, a low pH level is independent of hypoxia [54]. Both acid pH and high lactate levels activate endothelial cells to generate VEGF and fibroblast growth factor-β (FGF-β) [42,44]. Overproduction of lactate causes macrophages to generate VEGFA, which activates the initiation of both lymphatics and blood vessels [55,56,57,58]. VEGFA plays a key role with respect to vascular endothelial cells due to its associations with both VEGFR1 and VEGFR2. VEGFA activates the mitogenesis of endothelial cells and induces cellular migration. VEGF and FGF-β stimulate the proliferation and motility of endothelial cells and the budding of the vascular system [55,56,58]. An increase in lactate rates due to glycolysis stimulates HIF-1α in a positive feedback under normoxic conditions [57].

Lactate acid stimulates the production of metalloproteases and plasmin, disrupting the ECM, collagen and basal membrane [36,37,55]. Stored growth factors, including FGF-β, VEGFA and TGF-β, are released from the ECM [36,37,44,55]. TGF-β is a major controller of cancer cell migration [37,38].

Lactate activates the production of vascular growth factors by tumor cells, macrophages and fibroblasts [59]. Lactate activates the role of HIF-1α under normoxic conditions through several processes, regardless of the oxygen level [60,61,62,63]. Lactate stimulates the HIF-1α through the von Hippel–Lindau protein, a major protein for the destruction of HIF-1α. Lactate alters the role of the prolyl hydroxylase, which degrades HIF [44].

Numerous genetic mutations (including mutated genes that encode the colony stimulating factor (CSF) receptor), the epidermal growth factor receptor (EGFR), p53 and PTEN (phosphatase and TENsin homolog), result in HIF-1α stabilization and stimulate angiogenesis through the upward control of angiogenic factors [64,65,66,67,68]. Stimulation of HIF-1α increases the transcription of target genes, such as glucose transporters, monocarboxylate transporter-4 (MCT-4) and key proangiogenic effectors, such as VEGF [20,69]. HIF-1α also initiates both the glycolytic energy metabolism and angiogenesis and contributes to poor tumor prognosis [70,71]. Lactate causes excess collagen deposition [57,72] and plays a major role in angiogenesis through VEGF stimulation [73,74,75]. HIF-1α and lactate anion positively control the expression of MCT [76,77]. Lactate penetrates the tumor endothelial cells and the release of lactate by tumor cells via MCT-4 is sufficient to activate the process of angiogenesis [42]. In cancers, lactate also causes stimulation of HIF-1α independently of hypoxia by inhibiting the hydroxylation of the proline of HIF-1α [61]. Lactate overproduction in endothelial cells activates HIF-1α in a positive feedback loop in normoxia, resulting in the activation of proangiogenic genes, such as VEGFR2 [78]. VEGFR2 is a major modulator of the proangiogenic actions of VEGF [79]. Aerobic glycolysis-induced lactate stimulates the VEGF/VEGFR2 pathway in a manner independent of hypoxia [80]. Tumor cells surrounding the stroma have a high level of hyaluronan, which stimulates tumor growth and motility of tumor cells [81,82]. Hyaluronan refers to hyaluronic acid (HA) or hyaluronate and is a natural non-sulphate glycosaminoglycan (GAG) component found in the ECM of epithelial and neural tissues [83]. Hyaluronan has an important function in the development of embryonic tissues [84] and works to maintain the tissue homeostasis. Lactate stimulates the production of hyaluronan, which activates the angiogenic process [85,86]. Hyaluronan binds with membrane receptors, stimulates mitogenic genes and generates amoeboid movement [37]. HA oligomers are produced as HA degradation products by hyaluronidase (HYAl-1) [87]. High levels of lactate activate hyaluronidases to cause AH to be degraded into smaller fragments “in the form of oligomers” [85]. HA oligomers compete with native long-chain AH to bind with the HA CD44 receptor found in endothelial cells. HA oligomers stimulate the proliferation of endothelial cells [88] and initiate angiogenesis [89]. CD44-HA oligosaccharide interactions activate the production of matrix metalloproteinase-2 and-9 (MMP-2 and MMP-9), induce cell invasion, via ECM barriers, and generate vessel germination and growth [90,91].

## 3. The Key Role of the WNT/β-Catenin Pathway in Cancer Development under Normoxic Conditions 

The Canonical WNT/β-Catenin Pathway

The WNT name is derived from Wingless *Drosophila melanogaster* and its mouse homolog Int. The WNT/β-catenin pathway is involved in numerous signals and molecular pathways, including embryogenesis, cell proliferation, cell migration and cell polarity, apoptosis and organogenesis [92]. Nevertheless, the WNT/β-catenin pathway can be deregulated during numerous pathological states, such as inflammation, neurological disorders, metabolic diseases, tissue fibrosis and cancer mechanisms [93].

**Figure 1:**
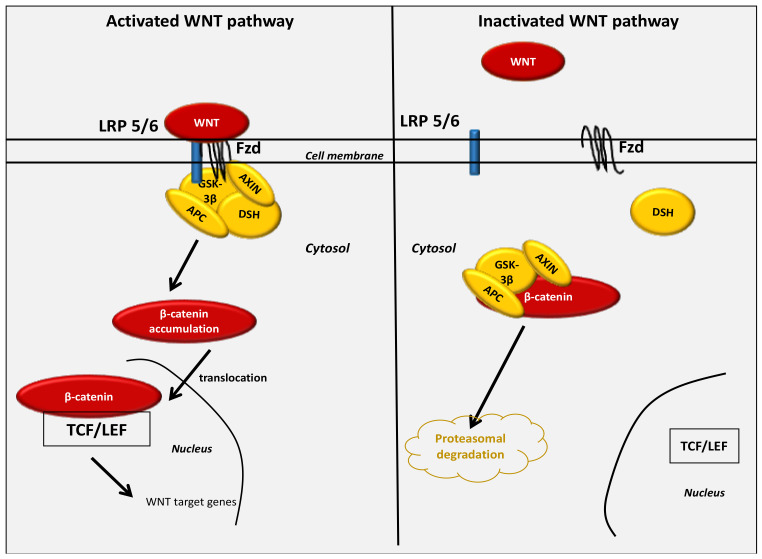
The activated and inactivated WNT/β-catenin pathway.

The WNT pathway belongs to the family of secreted lipid-modified glycoproteins [94] (Figure 1). WNT ligands are mainly secreted by both neurons and immune cells located in the central nervous system (CNS) [95]. Modulation of the WNT/β-catenin pathway involves metabolic pathways, embryonic development, cell fate and epithelial-mesenchymal transition (EMT). WNT pathway deregulation contributes to numerous neurodegenerative diseases (NDs) [96,97,98,99]. 

At the transcriptional level, WNT signaling is primarily mediated by a family of transcription factors known as the β-catenin/T-cell factor/lymphoid enhancer factors (TCF/LEF). The accumulation in the cytoplasm of β-catenin is generated by the destruction complex AXIN, tumor suppressor adenomatous polyposis coli (APC) and glycogen synthase kinase-3 (GSK-3β). In the absence of WNT ligands, the destruction complex enhances phosphorylation of the cytoplasmic β-catenin and induces its degradation in the proteasome. In the presence of the WNT ligands, β-catenin binds to Frizzled (FZL) and LDL receptor-related protein 5/6 (LRP 5/6) [100], thereby stopping the destruction complex and preventing the degradation of β-catenin in the proteasome. β-catenin translocates into the nucleus where it binds with the complex TCF/LEF. This in turn stimulates WNT targets [101,102,103].

GSK-3β is a major negative modulator of WNT/β-catenin signaling [104,105,106,107,108,109]. As an intracellular serine-threonine kinase, GSK-3β is a negative controller of the WNT pathway [110]. It is involved in the modulation of numerous kinds of pathophysiological signaling, such as the cell membrane pathway, cell polarity and inflammatory processes [111,112,113]. GSK-3β downregulates cytosolic β-catenin and stabilizes it, leading to the interruption of the nuclear migration of β-catenin. Inflammation is an age-related mechanism correlated with the stimulation of GSK-3β activity and a decrease in WNT/β-catenin pathway activity [114]. 

## 4. The WNT/β-Catenin Pathway Stimulates Cancer Metabolic Reprogramming under Normoxic Conditions

### 4.1. Interactions between the Canonical WNT/β-Catenin Pathway and the Warburg Effect 

**Figure 2:**
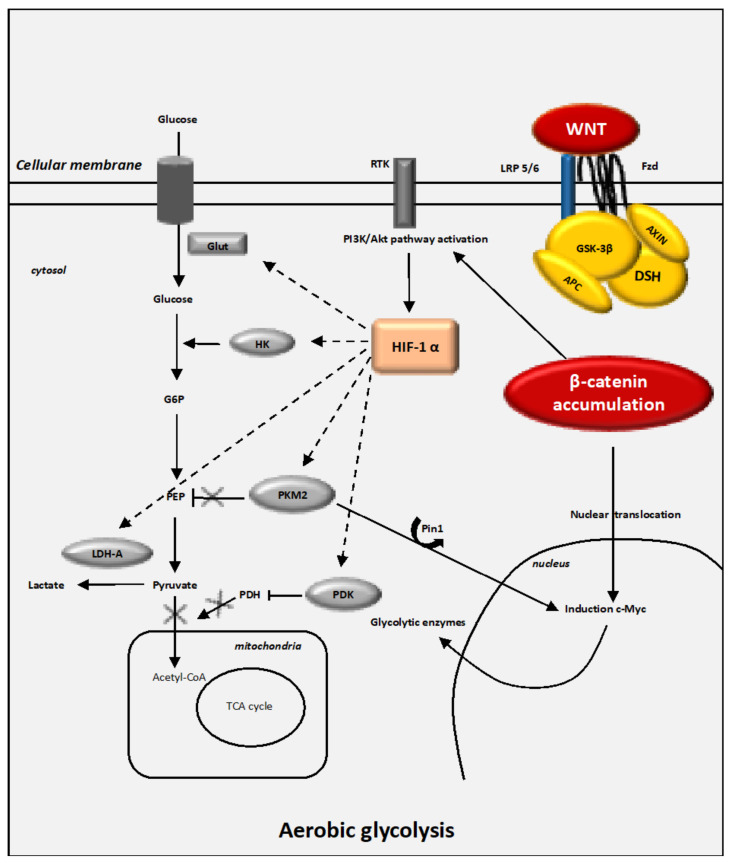
The WNT/β-catenin pathway and the Warburg effect. WNT ligands bind with the Frizzled/LRP 5/6 receptor complex, leading to LRP phosphorylation of the AXIN/APC/GSK-3β complex. β-catenin accumulates in the cytoplasm to translocate to the nucleus. The WNT-process transcription activity is then stimulated. MCT-1 enhances lactate release out of the cell. The stimulation of the PI3K/Akt pathway is associated with the activation of the glucose process. Increased levels of aerobic glycolysis are associated with higher lactate production and a decrease in mitochondrial respiration. HIF-1α activation leads to the cytoplasmic pyruvate being shunted into lactate by MDH-A induction. The pyruvate cannot then be fully converted into acetyl-CoA and enter the TCA cycle. The entry of glucose into the TCA cycle is modulated by the pyruvate dehydrogenase complex (PDH). A decrease in PDH inhibits the activity of the mitochondria in several cancers [115]. In tumor cells, the activation of the WNT/β-catenin pathway enhances aerobic glycolysis [116]. PDK-1 is activated in vitro and in living colon tumor cells [117] and there is a decrease in the conversion of pyruvate into acetyl-CoA in mitochondria [118]. PDK-1, LDH-A and MCT-1 are stimulated in cancer cells [57,119]. c-Myc and cyclin D1 also stimulate aerobic glycolysis [120]. The activation of c-Myc and PI3K/Akt signaling leads to the stimulation of HIF-1α activity, which inhibits glucose entry into the TCA cycle [121,122]. The stimulation of HIF-1α increases the expression of Glut, HK, PK, PDK-1 and LDH-A [20,123,124]. Glut allows the entry of glucose into the cytoplasm where glycolysis ensues. HK phosphorylates glucose during the first stage of glycolysis. PK controls the production of pyruvate during the last stage of glycolysis. PDH stimulates the entry of pyruvate into the oxidative phosphorylation process and thus the tricarboxylic acid (TCA) cycle within the mitochondria. Activation of HIF-1α stimulates PDK-1, inactivating PDH. The stimulation of LDH-A leads to the conversion of pyruvate into lactate. Abbreviations: GSK: glycogen synthase kinase, DSH: disheveled, APC: adenomatous polyposis coli, HIF: hypoxic inducible factor, RTK: tyrosine kinase receptor, Glut: glucose transporter, HK: hexokinase, PKM2: pyruvate kinase M2, LDH: lactate dehydrogenase, PDK: pyruvate dehydrogenase kinase, TCA: tricarboxylic cycle.

Glucose is the main source of energy for cells. Glucose is metabolized to generate ATP (as energy) by cytoplasmic glycolytic mechanisms and oxygen-dependent mitochondrial processes (Figure 2). 

Tumor cells are dependent on glycolysis for their primary source of energy [125], with activation of the glycolytic metabolism [126]. Instead of primarily using oxidative phosphorylation (OXPHOS), cancer cells use less efficient glycolysis for ATP production. However, some cancers have the ability to produce ATP in an OXPHOS-dependent manner, such as cancer stem cells [127]. Activation of the intracellular concentration of lactate in tumor cells is associated with a progressive decrease in TCA cycle activity [128].

### 4.2. Interactions between the WNT/β-Catenin Pathway and STAT3 Pathway 

The STAT3 (signal transducer and activators of transcription 3) belongs to the family of cytosolic transcription factors that allow the activation of nuclear pathways [129]. The STAT3 pathway plays an important role in cell mechanisms, including differentiation, proliferation, apoptosis and angiogenesis [130]. A stimulated STAT3 pathway is observed in several cancers [131]. The STAT3 pathway induces tumorigenesis by the stimulation of genes encoding cell-cycle regulators (cyclin D1, c-Myc) and also stimulates VEGF activity [132]. 

**Figure 3:**
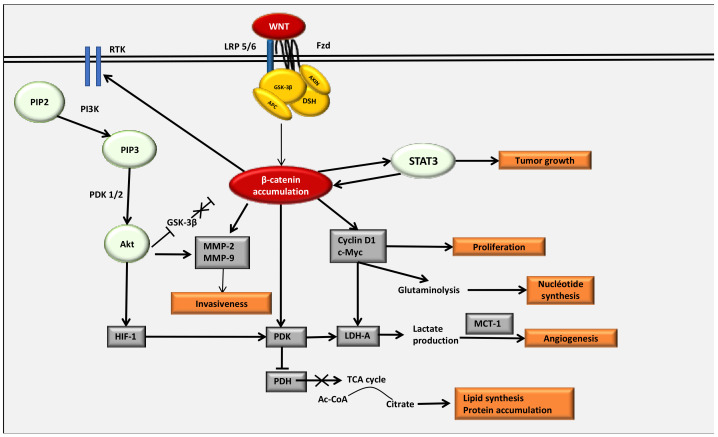
The WNT/β-catenin pathway and its different actions in cancer processes. The WNT plays a major role in cancer processes by interacting with the STAT3 pathway, leading to tumor growth, by stimulating cyclin D1 and c-Myc, responsible for cell proliferation and then for the glutaminolysis mechanism responsible for the nucleotide synthesis. β-catenin directly activates RTK, which in turn stimulates the PI3K/Akt pathway leading to the inactivation of the GSK-3β, a major inhibitor of β-catenin. Activated Akt stimulates MMP-2 and MMP-9, which are responsible for the invasiveness. The double action of β-catenin and the PI3K/Akt pathway for the activation of the HIF-1α enhances the stimulation of the PDK, which in turn inactivates the PDH and stops the TCA cycle. The TCA cycle arrest leads to lipid synthesis and protein accumulation in cancers. Moreover, LDH-1 is directly responsible for the production of lactate and its translocation over the cell by MCT-1, which in turn stimulates angiogenesis. Abbreviations: GSK: glycogen synthase kinase, HIF: hypoxic inducible factor, RTK: tyrosine kinase receptor, LDH: lactate dehydrogenase, PDK: pyruvate dehydrogenase kinase, TCA: tricarboxylic cycle, MCT: monocarboxylate transporter.

A STAT3 phosphorylated by IL-6 or LIF (leukemia inhibitory factor) is associated with activation of HIF-1α in normoxia [133]. The β-catenin/TCF4 complex directly interacts with STAT3 signaling [134]. STAT3 is essential for mesenchymal transformation and tumor aggressiveness [135] and is considered as a downstream pathway of mTOR signaling [136]. STAT3 and its downstream Notch receptor operate in several cellular and molecular mechanisms such as proliferation, differentiation and apoptosis [137]. The decrease in STAT3 activity inhibits cancer cell growth, invasion, migration, cell-cycle progression and differentiation [138] (Figure 3).

### 4.3. Interaction between the WNT/β-Catenin Pathway and the PI3K/Akt Pathway 

The receptor tyrosine kinase (RTK) and the EGFR are enhanced in cell division, migration, adhesion, differentiation and apoptosis [139,140]. EGFR interacts with the PI3K/Akt/STAT pathways to control proliferation, differentiation and cell survival [141,142]. PI3K is directly stimulated by EGFR. PI3K converts PIP2 into PIP3 [143,144]. The role of PI3K is inhibited by PTEN, which converts PIP3 back to PIP2 [145]. PI3K stimulates the Akt pathway by phosphorylating it [146]. The activated Akt pathway decreases the activity of GSK-3β and allows the nuclear translocation of β-catenin [143]. The PI3K/Akt pathway controls β-catenin stability, nuclear translocation, transcriptional activity and the expression of its downstream genes, including Cyclin D1 and c-Myc [144]. 

The PI3K/Akt pathway plays a key role in the control of cellular metabolism, growth, proliferation and cell survival [147,148]. The WNT/β-catenin pathway directly activates RTK in tumor cells [149] (Figure 3). Decreased β-catenin leads to a reduction in EGFR expression as well as in the Akt pathway and its downstream genes [150]. The WNT/β-catenin pathway and the PI3K/Akt pathway control each other in a positive feedback loop. PI3K produces PIP3, binding with PDK-1 through a pleckstrin homology domain (PH). The activation of PDK-1 phosphorylates and then stimulates the Akt pathway [151]. The mechanistic target of rapamycin (mTOR) is one of the main markers of tumor growth. The kinase mTOR acts through two different proteins, i.e., mTORC1 and mTORC2, which are distinct with respect to the composition of their subunits, upstream inputs, downstream substrates and their roles [148,152].

PI3K activates mTORC1 through a dependent Akt pathway. mTORC1 decreases the tuberous sclerosis complex (TSC) [153,154]. TSC phosphorylation decreases its GTPase-activating protein (GAP) activity for the small GTPase Ras homolog enriched in brain (Rheb). This phenomenon leads to a decrease in Rheb and the activation of mTORC1 [155,156]. Rheb is a major positive marker of mTORC1 [157,158,159] through direct binding with mLST8, the kinase domain of mTOR, [157,160]. By a mTORC1 negative loop, Rheb can downregulate PI3K and mTORC2 pathways [161]. mTORC2 can also stimulate the Akt pathway [162]. Nevertheless, mTORC2 activity is enhanced in a PI3K-dependent manner [163]. The PI3K/Akt pathway can stimulate mTORC2 activity by activating IKKα [164]. Stimulation of PI3K/Akt signaling is associated with the activation of mTORC1 [165], following its dissociation from PRAS40.

### 4.4. The Key Enzymes Involved in Metabolic Reprogramming in Cancer Cells

#### 4.4.1. HIF-1α 

HIF-1α expression in human bodies enhances angiogenesis by transcriptionally stimulating several angiogenic genes and their receptors, including VEGF [166]. HIF-1 is a heterodimeric nuclear transcription factor consisting of two compounds: HIF-1α and HIF-1β. The action of HIF-1α is modulated by changes in local oxygen rates. Under normoxia, HIF-1α is destroyed by HIF prolyl hydroxylases [167,168].

Activation of HIF-1α leads to the activation of gene transcription encoding several glycolysis enzymes (i.e., LDH-A, PKM2 and PDK-1) [169,170,171] (Figure 2 and Figure 3). HIF-1α is stimulated in several oncogenic processes even under normoxic conditions [57,58,172,173]. The transcriptional activity of HIF-1α is stimulated by mTOR even under normoxia by 4E-BP1 and STAT3 [133,174,175,176,177,178]. mTOR-mediated HIF-1α induction can mimic the action of hypoxia, leading to the glycolysis mechanism [123].

#### 4.4.2. Glut-1 and HK2 

Glucose-transporters, Glut-1 and Glut-3, have their main roles in the insulin-sensitive homeostasis of glucose transport [179]. Glut-1 expression is stimulated in cancer processes [180]. HIF-1α stimulates the glycolytic flux by activating the glucose uptake and its metabolism by directly activating both Glut-1 and Hexokinase 2 (HK2) [118,181,182] (Figure 3). HIF-1α stimulates the expression of Glut-1 to increase the intracellular glucose levels, which favor glycolysis and tumor development [183,184,185].

HK2 activation leads to the transformation of glucose into glucose-6-phosphate. HK2 is stimulated by both c-Myc and HIF-1α [121,124,186]. High rates of HK2 lead to the development of cancers [187]. However, the downregulation of HK2 activity in cancers could prevent the aerobic glycolysis mechanism, leading to a reduction in tumor growth [188]. 

#### 4.4.3. PKM2 

The final step of glucose metabolism is the conversion of phosphoenolpyruvate and adenosine diphosphate (ADP) into both ATP and pyruvate. The enzyme pyruvate kinase (PK) is responsible for this phenomenon. Four isozymes of PK are defined: PKL, PKR, PKM1 and PKM2 [189]. PKM2 is overstimulated in cancers [190,191]. Under high glucose levels, PKM2 enters into the nucleus through the activity of peptidyl-prolyl isomerase 1 (Pin1) [192] (Figure 3). Pin1 is stimulated in cancer processes to inactivate tumor growth inhibitors [193,194]. Pin1 is directly stimulated by EGFR to generate the nuclear translocation of PKM2 [195]. Under normoxic conditions, mTOR activates PKM2 activity by stimulating HIF-1α [123]. Thus, nuclear PKM2 can target β-catenin leading to c-Myc-mediated expression of glycolytic enzymes [195].

#### 4.4.4. PDK-1 

PDK-1 acts as a major modulator of the glycolysis metabolism by directly phosphorylating the PDH complex to downregulate the mitochondrial conversion of pyruvate to acetyl-CoA [196]. PDK-1 controls the activity of PDH by phosphorylating three serine residues in the α subunit of PDH-E1 [196], resulting in the inhibition of its activity (Figure 3). The stimulation of PDK is associated with the shunt of pyruvate away from the mitochondria to downregulate the flux through the TCA cycle. This action inhibits the release of NADH from the electron transport chain [115]. HIF-1α directly stimulates the activity of PDK-1 to inhibit the conversion of pyruvate into acetyl-CoA [115].

#### 4.4.5. LDH-A 

LDH is a tetrameric enzyme that belongs to the 2-hydroxy acid oxidoreductase family. LDH stimulates the levels of both the inter-conversion of pyruvate to lactate and the nicotinamide adenine dinucleotide (NADH) into NAD+ [197]. Four LDH subunits are defined: LDH-A, LDH-B, LDH-C and LDH-D. LDH-A is the main enantiomer observed in vertebrates. LDH-A plays a number of major roles in cancer cells, including gene transcription, angiogenesis, tumor migration and tumor evasion of immune response [37,38,198,199,200]. In cancer cells, the stimulation of LDH-A activates the lactate production [201]. LDH-A is mainly stimulated by both HIF-1α and c-Myc [202,203,204,205] (Figure 3). Under normoxic conditions and in a positive interplay, LDH-A can stimulate HIF-1α expression to enhance lactate production [61,206]. Under normoxic conditions, LDH-A is also stimulated by c-Myc to promote HIF-1α stabilization [200]. Moreover, the stimulation of LDH-A leads to the enhancement of VEGF [207,208]. 

Independently of hypoxia, acidic extracellular pH, caused by the increased lactate production, can upregulate IL-8 and VEGF [48,53,54,209,210,211]. In cancer cells, VEGF expression is modulated by pH and tissue pO2 [54]. Hypoxia and acidic pH present no synergistic actions on the transcription of VEGF [54]. 

## 5. Definition of the Autophagy Mechanism

It is widely known that autophagy is damaged in cancer processes. Autophagy represents a major mechanism in preserving stem cell homeostasis by finely tuning stem cell maintenance and differentiation [212,213]. In physiological cells, autophagy can act as a tumor-suppressive process through the maintenance of cell homeostasis, whereas in tumor cells, autophagy can exert either tumor-promoting or tumor-suppressing actions. It is still a subject of debate as to whether autophagy activation or diminution could provide the most promising approach for future cancer therapies [214].

Autophagy (from the Greek word for “self-eating”) is a phenomenon that depends on energy, and one that is highly regulated and conserved. Autophagy is responsible for the sequestration and recycling of redundant or altered cell organelles, long-lived proteins and even pathogens [215]. A basal rate of autophagy plays a major role in many cell types, maintaining cell homeostasis under normal conditions. However, its alteration can appear during development and results from several types of stress, including nutrient deprivation, DNA damage, hypoxia and oxidative metabolism [215].

Three types of autophagy may be identified at the cellular level: micro-autophagy, macro-autophagy and chaperone-mediated autophagy (CMA) [216]. 

Micro-autophagy follows the direct invagination of the lysosomal membrane, which forms an autophagic body and causes the degradation of the sequestered part of the cytosol [216]. The CMA involves a related thermal shock and a complex of other chaperones that recognize cytosolic target proteins with a KFERQ pattern. The CMA acts with the membrane protein associated with type 2a lysosome (LAMP2A) and directly brings the cargo to the lysosomal membrane [217,218]. The main form of autophagic degradation, that of macro-autophagy, occurs through multi-level phenomena beginning with the engulfment of cytosolic components in a crescent-shaped double membrane structure called the phagophore [219,220]. 

Autophagy can be investigated at various stages: induction and initiation of autophagy, nucleation of the phagophore, expansion of the phagophore, targeting of cargo, completion of autophagosomes and fusion with lysosomes, and degradation of content [215]. Autophagy can have a dual role. During stress, autophagy induces cytoprotective effects that prolong cell survival, while over-stimulation of cell survival/ cytoprotective effects can be harmful to the cell and induces apoptosis [221]. Numerous studies have demonstrated the involvement of deregulated autophagy in various pathological processes, such as cancer [222], neurodegenerative diseases [223], liver diseases [224], aging [225] and viral infections [226]. 

## 6. Autophagy in Cancer

As a crucial compound of cell defense mechanisms, autophagy has an important role in the destruction mechanism of damaged organs, dysregulated proteins and metabolite recycling in order to maintain the stability of the internal environment [227]. In cancers, autophagy has a dual role in both the promotion and suppression of tumors. Basal autophagy is weak and almost considered as a process of cancer suppression in the early stages of carcinogenesis through inhibition of ROSs, DNA and tissue damage, inflammation and genome instability [228]. Thus, the lack of key genes in autophagy may be the cause of carcinogenesis processes. The deletion of ATG5 and ATG7 contributes to the development of benign liver adenomas from autophagy-deficient hepatocytes via mitochondrial swelling and oxidative stress [229]. The BECN1 (Beclin 1) gene linked to autophagy is essential in the formation of the phagophore. The loss of the Beclin 1 gene linked to autophagy may contribute to the development of spontaneous tumors and can lead to an increase in cell proliferation in different types of tumors [230,231]. Autophagy, as a cancer suppressor, is also associated with a replicative seizure [232,233]. Replicative crisis is the final barrier before tumor development, leading to mitotic delay, amplified telomere deprotection and cell death. Autophagy also contributes to the tumor suppression crisis. The loss of autophagy stimulates the continuous proliferation and accumulation of genomic instability, a process essential to the onset of cancer [234]. Autophagy is considered to be a tumor suppressor because the process leads to the elimination of the oncogenic proteins involved in oncogenesis. BCR-ABL1, an oncogene protein responsible for the progression of leukemia, is degraded by autophagy [235].

However, while autophagy slows down the tumor initiation process, other studies have suggested that decreased autophagy may contribute to the tumor initiation process. Genetic ablation of essential autophagy genes in genetically modified mouse models for tumors has revealed the major role of autophagy in promoting malignant tumors [236,237]. Autophagy also promotes angiogenesis, metastases and invasion during tumorigenesis. Autophagy stimulates VEGFA through the janus kinase-2/signal-transducer and activator transcription-3 (JAK2/STAT3) and plays a major role in angiogenesis [238]. In parallel, the triggered autophagy could activate the Hippo YAP/TAZ signaling and stimulate cell invasion and tumor migration [239].

## 7. Interplay between Glycolysis and Autophagy in the Cancer Process

During tumor growth, cancer cells use metabolic reprogramming to maintain growth, survival, proliferation and migration processes. To meet different metabolic needs, autophagy is modified. Currently, the relationship between autophagy and glycolytic metabolism is subject to extensive investigation. Glucose is the major source of nutrients for the production of cellular energy and a constant supply of glucose is required for the production of ATP. As a major biological mechanism, glycolysis is consubstantial with autophagy. Metabolic reprogramming induces cancer progression to regulate autophagy. Thus, glycolysis controls autophagy and participates fully in the survival of tumor cells. 

When the glycolytic process is decreased, as in the context of aerobic glycolysis, autophagy is improved and becomes a driving force for oxidative phosphorylation to support the production of ATP [240]. Pyruvate supplementation prevents RSL3-induced cell death, indicating that the dysfunction of glycolysis can cause autophagic cell death in glioma cells [241]. Similarly, the stability of EGFR, mediated by aerobic glycolysis, is essential for tumor survival. The inhibition of ATP production leads to the activation of ROS-mediated c-Jun N-terminal kinase (JNK) and thus to the degradation of EGFR via inhibited autophagy [242]. Aerobic glycolysis regulates autophagy and plays a major role in the chemo-resistance of cancers. HK2 confers cisplatin resistance in ovarian cancer cells by increasing ERK1/2 phosphorylation and autophagic activity. Stopping autophagy through the autophagy inhibitor 3-methyladenine (3-MA) sensitizes cisplatin-resistant ovarian cancer cells [243]. Several studies have shown that in the most glycolytic dependent cells, autophagy is usually triggered by the accumulation of adenosine monophosphate (AMP) (via increased AMP/ATP ratio) and the stimulation of AMPK [244]. Thus, metabolic reprogramming caused by aerobic glycolysis stimulates autophagy, also induced by OXPHOS, and thus stimulates intracellular ROSs [245]. However, the mechanism for controlling aerobic glycolysis on autophagy is still not well understood. One interesting avenue of research is to examine the role of glycolytic enzymes in the functioning of the WNT pathway and its targets.

### 7.1. Key Role of the WNT/β-Catenin Pathway in Cancer Development through Interaction with Autophagy

**Figure 4:**
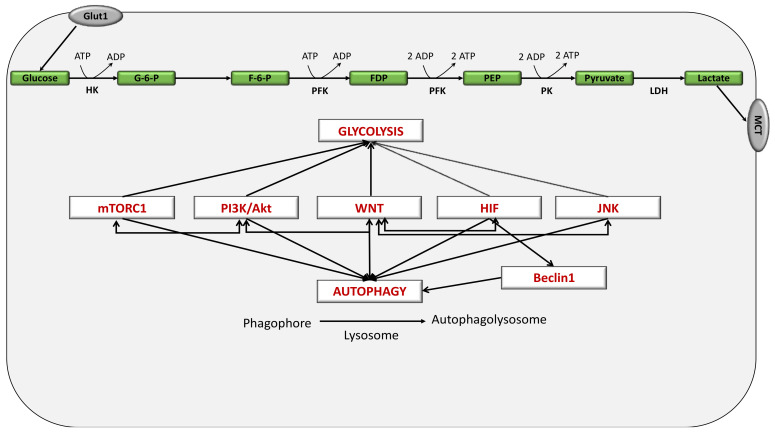
Interplay between the WNT/β-catenin pathway, aerobic glycolysis and autophagy. The interactions between WNT, PI3K/Akt pathway, mTORC1, HIF and JNK result in stimulating both glycolysis and autophagy, involving several glycolytic enzymes (including, glucose, G6P, F6P, FDP, PEP, pyruvate producing lactate over the cell by MCT). Abbreviations: G-6-P: glucose 6 phosphate, F-6-P: fructose 6 phosphate, FDP: fructose diphosphate, PFK: phosphofructokinase, PEP: phosphoenolpyruvate, JNK: Jun N-terminal kinase, HIF: hypoxic inducible factor, mTORC1: mechanistic target of rapamycin C1.

The interaction between autophagy and distinct signaling, such as that associated with the WNT/β-catenin, SHH and TGF-β pathways, has a vital role in several cellular mechanisms [246] (Figure 4). 

Numerous studies have shown that activated WNT/β-catenin is a negative controller of autophagy based on the evidence that high rates of autophagy are not compatible with cellular proliferation and survival enhanced by the WNT/β-catenin pathway [247]. Activated WNT/β-catenin diminishes the expression of Beclin 1, an inducer of autophagic flux [248]. Moreover, the activation of autophagy can decrease WNT/β-catenin pathway activity through the degradation of DVL [249] and β-catenin [247]. Feedback processes between the WNT/β-catenin pathway and autophagy have been shown at distinct rates [215]. β-Catenin contains a LC3 interacting region (W/YXXI/L motif) that reveals a direct target of selective autophagy destruction via the LC3 binding site [215]. Moreover, activated autophagy leads to a decrease in WNT signaling through the autophagic degradation of β-catenin [247]. β-catenin can act as a gene repressor [250]. 

Thus, the WNT/β-catenin pathway exercises a negative control on autophagy. The nuclear accumulation of β-catenin leads to the inhibition of the p62/SQSTM1 promoter, which enhances autophagy diminution [250]. Conversely, the decrease in WNT/β-catenin pathway activity leads to the stimulation of p62/SQSTM1 expression and to the nuclear accumulation of the TFEB transcription factor, inducing the autophagy process [251]. 

DVL is involved in the regulatory feedback process between autophagy and the WNT/β-catenin pathway [252]. DVL is a selective target of destruction by a direct interaction with p62 and LC3. Under stress conditions, the von Hippel–Lindau protein (pVHL, an E3 ligase) triggers the ubiquitination of DVL, whereas hVHL is negatively regulated by GSK-3β. p62 recognizes ubiquitinated DVL by its ubiquitin-associated domain and binds with LC3. DVL has an affinity with the LC3 interacting region (LIR). p62 binds with LC3 through LIR, which can directly target LC3, directing DVL degradation into the autophagosome [249]. Thus, the non-inhibition of GSK-3β activity by DVL leads to β-catenin phosphorylation, ubiquitination and then a diminution in WNT/β-catenin pathway activity. DVL is phosphorylated by active ULK1 to become inactive [253]. Thus, following autophagy induction, DVL is inhibited and the WNT/β-catenin pathway is attenuated [254,255]. GSK-3β is involved in mTOR signaling where tuberous sclerosis 2 (TSC2) is phosphorylated and stimulated by GSK-3β in association with AMPK [256]. Activation of TCS2 leads to mTOR diminution and thus to autophagy induction. The PI3K/Akt pathway catalyzes the phosphorylation of GSK-3β [257]. During autophagy induction, a positive feedback process is stimulated where DVL (a GSK-3β inhibitor) is damaged by LC3 interaction and derepressed GSK-3β, leading to autophagy by TSC2 phosphorylation [258]. GSK-3β represses the WNT/β-catenin pathway and induces autophagy by phosphorylating TCS2. However, stimulated TSC2 decreases mTORC1, enhancing β-catenin stabilization [215]. The interplay between DVL–PI3K/ –WNT/β-catenin pathway and the development of autophagy could provide the basis for cancer initiation [259].

Conversely, in vitro studies have shown that the WNT/β-catenin pathway and autophagy can be stimulated at the same moment [260]. Thus the WNT/β-catenin pathway was found to be involved in autophagy-induced glycolysis in hepatocarcinomatous cell lines [261]. Moreover, the WNT3a ligand activates autophagy in squamous cell carcinoma of the head and neck, sensitizing cells to radiotherapy [262]. 

### 7.2. The Key Role of the WNT/β-Catenin Pathway in Cancer Development through Interaction with Glutaminolysis 

In parallel to the shift toward the Warburg effect, tumor cells rely on the provision of bulk quantities of amino acids as major enhancers for cellular survival and growth, in order to complete the TCA cycle [263]. Stimulation of the glutamine uptake is well-known in different cancers, such as breast cancer [264,265]. Nevertheless, glutaminolysis should be cancer-subtype-dependent. Triple-negative breast cancers are mainly dependent on glutamine so that therapies using glutamine-targeting drugs could provide the most effective treatment [266]. Glutamine has a major role in cell proliferation, survival and migration [120,267]. It undergoes glutaminolysis, a mechanism by which it is converted to glutamate and α-ketoglutarate to replenish the TCA cycle, enhance protein synthesis and generate GSH. α-Ketoglutarate can supplement the Warburg effect as it can be converted to malate and then to pyruvate [268]. Glutaminolysis is an energy pathway that is used by tumor cells to supply nitrogen to sustain their rapid division and energy requirements [269]. 

**Figure 5:**
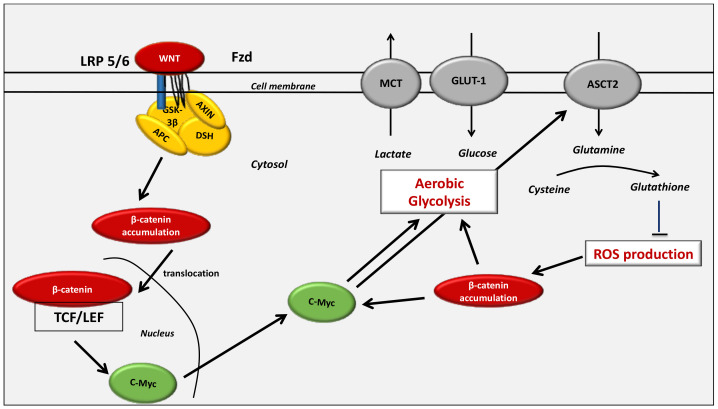
Interplay between the WNT/β-catenin pathway, aerobic glycolysis and glutaminolysis. c-Myc has been observed to play a role in the activation of genes involved in glutamine metabolism, including the glutamine transporter ASCT2 (or SLC1A1) and glutaminase [271]. Moreover, β-catenin can control glutamine metabolism and glutaminolysis by directly acting on c-Myc [272]. Aerobic glycolysis, directly modulated by the WNT/β-catenin pathway stimulates the different receptors of the glycose pathway and activates the MCT (monocarboxylate transporters) to translocate the lactate over the cell. The process of glutaminolysis, indirectly activated by the aerobic glycolysis and β-catenin signaling leads to the down-production of ROS, which in turn activates the β-catenin. Abbreviations: MCT: monocarboxylate transporter, Glut-1: glucose transporter 1, ASCT2: glutamine transporter 2, ROS: reactive oxygen species.

The WNT/β-catenin pathway plays an important role in glutamine metabolism. Research has shown that β-catenin target genes are involved in glutamine uptake and metabolism [270]. While the exact process by which WNT/β-catenin interacts with glutaminolysis is still unclear, c-Myc could play a key role [271] (Figure 5). 

Although it has been suggested that the NF-κB pathway is a modulator of glutaminolysis in breast tumor cells, the role of the WNT/β-catenin pathway in this control is still unclear [267].

GSH, a direct product of glutamine metabolism, plays a key role in the chemoresistance of tumor cells [273] and cancer stem cells (CSCs) [274]. GSH is directly associated with the WNT/β-catenin signaling [275]. The role of GSH is essential in CSC chemoresistance, as it has been observed that CD44+, a CSC surface marker, in association with glutamine-cysteine transporter, enhances GSH synthesis [274]. GSH protects the cells against OS by inhibiting ROS production [276]. Therapy with the GSH inhibitor can downregulate tumor growth in mouse models [277]. Deprivation of glutamine in CSCs leads to a decrease in GSH and an increase in β-catenin phosphorylation, leading to the inhibition of the WNT/β-catenin pathway. The regulation of glutamine in stem-like cancer cells can occur through ROS-mediated β-catenin phosphorylation and proteasomal degradation [275]. Thus, the production of GSH may participate in the decrease of the CSC by decreasing WNT/β-catenin pathway activity. Thus, c-Myc is not damaged by a glutamine withdrawal in these types of cancer stem cells, indicating no feedback loop in the c-Myc control of glutamine metabolism [275]. 

## 8. Interaction between the WNT/β-Catenin Pathway and the Hippo Pathway: The Link between Glycolysis and Glutaminolysis 

Yes-associated protein 1 (YAP) / transcription coactivator and PDZ-binding motif (TAZ) are transcription co-activators of the Hippo kinase complex [278]. The Hippo pathway modulates organ size and cellular regeneration and involves several compounds such as serine/threonine protein kinases (MST1/2), MOB kinase activator 1 (MOB1), Salvador (SAV) and the serine/threonine protein kinase-(LATS1/2). When the kinase Hippo is in the stimulated stage, YAP and TAZ are phosphorylated and involve a phosphodegron. YAP/TAZ can be sequestered into the cytoplasm or destroyed by β-TrCP proteins. The stimulated Hippo pathway results in the phosphorylation of YAP and the reduction of β-catenin levels.

There is interplay between the WNT/β-catenin signaling and the YAP/TAZ signaling. The YAP/TAZ pathway is responsible for WNT-induced biological mechanisms [279,280], while β-catenin stabilization is responsible for the nuclear TAZ accumulation and its transcriptional activity [279]. In cancer cells, the main part of the transcriptional activity of the WNT target genes is TAZ-dependent. This means that the YAP/TAZ pathway is an integral compound of the β-catenin destruction complex [280]. 

**Figure 6:**
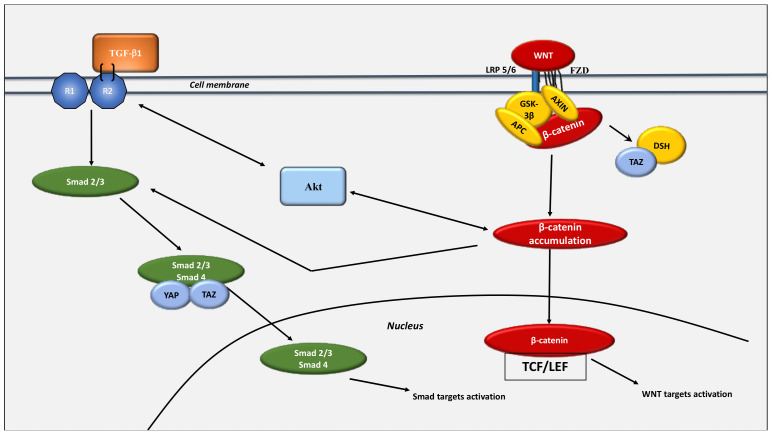
Interaction between the WNT/β-catenin pathway and the Hippo pathway. Crosstalk between the YAP/TAZ and TGF-β pathways is observed. TAZ interacts with Smad2/Smad3 [285] to induce the nuclear transfer of Smad2/3 and then to transcribe the PAI-1 and Smad target genes. In the cytosol, TAZ is bound to the WNT/β-catenin signaling by a TAZ/β-catenin complex [279]. WNT stimulation is associated with the inhibition of the β-catenin destruction complex, resulting in cytosolic β-catenin accumulation and its nuclear translocation for stimulating the different WNT targets. Following WNT activation, TAZ decreases the DSH phosphorylation and liberates it from the destruction complex. The destruction complex is destroyed because YAP and TAZ dissociate from the complex. Following TGF-β1 stimulation, AXIN leads to the enhancement of the tail-phosphorylation of Smad2/3. The stimulated Smad2/3-Smad4 complex interacts with TAZ and YAP and translocates to the nucleus for the stimulation of the Smad targets. TGF-β1 leads to Smad2/3 and PI3K/Akt signaling overactivation. The nuclear translocation of β-catenin blocks the degradation of TAZ. This relationship of TAZ with the WNT/β-catenin pathway is independent of its role as a mediator of the Hippo pathway. When WNT/β-catenin signaling activity is decreased, the cytoplasmic YAP/TAZ specifically interacts with AXIN [280].

APC binds with SAV1 and LATS1, which are upstream controllers of YAP/TAZ. The modulation of the YAP/TAZ pathway by APC is a major reason for intestinal tumor progression. Furthermore, DVL is required for the nucleocytoplasmic shuttling of YAP [281]. WNT activates the YAP/TAZ pathway by FZD overexpression [282]. YAP can also be a direct transcriptional target of the WNT/β-catenin pathway in tumor cells [283]. WNT3a promotes the TGF-β1 pathway through Smad2 phosphorylation [284] (Figure 6).

Activation of YAP/TAZ leads to stimulation of glutamine levels, to the enhancement of nucleotide synthesis, and to the promotion of glutamine synthetase (GS) expression [286]. The stimulation of YAP/TAZ modulates metabolic enzyme expression, including that of glutaminase (GLS1), to control both glutaminolysis and glycolysis [287]. The diminution of YAP/TAZ decreases both the production of lactate and the extracellular lactate/pyruvate ratio. YAP/TAZ knockdown also shunts the actions of a stiff extracellular matrix on intracellular glutamine, glutamate and aspartate [288]. However, high rates of YAP pathway activity stimulate the extracellular lactate levels and the lactate/pyruvate ratio, diminish glutamine expression and stimulate glutamate and aspartate expression [287]. 

The stimulation of YAP/TAZ leads to the enhancement of glycolysis in cancer cells. YAP-5SA activates YAP [289], stimulates the glucose uptake and the production of lactate in cells and also presents lower pH values [290]. Activated YAP involves the transcription activity and expression of GLUT3. YAP leads to the promotion of glycolysis in cancer cells through the direct control of GLUT3 transcription [290]. YAP is enhanced by the forkhead box protein C2 (FOXC2). The activation of YAP positively regulates the expression of HK2 at both mRNA and protein levels [291]. FOXC2 can interact through a crossbridge to bind with YAP to both stimulate HK2 and enhance glycolysis in cancer cells [291]. The YAP/TAZ pathway is a major metabolic process in the control of glycolysis [287].

## 9. Conclusions

The WNT/β-catenin pathway is over-activated in several cancers and plays a major role in proliferation, invasion, apoptosis and angiogenesis. Moreover, the nuclear β-catenin levels are associated with malignancies. Hypoxia is a well-known process that activates HIF-1α to promote the glycolysis metabolism. The WNT/β-catenin pathway stimulates the PI3K/Akt and STAT3 pathways and the transduction of several target genes, including c-Myc, which can stimulate HIF-1α in a hypoxia-independent manner. In cancers, the stimulation of the WNT/β-catenin pathway leads to many glycolytic enzymes that in turn induce metabolic reprogramming (the Warburg effect) in association with autophagy modification. This metabolic reprogramming leads to the production of lactate, as the primary alternative of ATP, at all oxygen rates, even under normoxia. An increase in lactate production is correlated with the modification of the tumor microenvironment, the Warburg effect, autophagy and glutaminolysis, leading to tumor growth under normoxic conditions.

## Abbreviations

APC	Adenomatous polyposis coli
CK1	casein kinase 1
COX-2	Cyclooxygenase-2
FZD	Frizzled
GSK-3β	Glycogen synthase kinase-3β
LRP 5/6	Low-density lipoprotein receptor-related protein 5/6
MAPK	Mitogen-activated protein kinases
NF-κB	nuclear factor κB
NOX	NADPH oxidase
PPARγ	Peroxisome proliferator-activated receptor gamma
PI3K-Akt	Phosphatidylinositol 3-kinase-protein kinase B
ROS	reactive oxygen species
TCF/LEF	T-cell factor/lymphoid enhancer factor
TNF-α	tumor necrosis factor alpha.
